# Transcriptomic convergence despite genomic divergence drive field cancerization in synchronous squamous tumors

**DOI:** 10.3389/fonc.2024.1272432

**Published:** 2024-06-13

**Authors:** Qiu Xuan Tan, Nicholas B. Shannon, Weng Khong Lim, Jing Xian Teo, Daniel R. Y. Yap, Sze Min Lek, Joey W. S. Tan, Shih Jia J. Tan, Josephine Hendrikson, Ying Liu, Gillian Ng, Clara Y. L. Chong, Wanyu Guo, Kelvin K. N. Koh, Cedric C. Y. Ng, Vikneswari Rajasegaran, Jolene S.M. Wong, Chin Jin Seo, Choon Kiat Ong, Tony K. H. Lim, Bin Tean Teh, Oi Lian Kon, Claramae S. Chia, Khee Chee Soo, N. Gopalakrishna Iyer, Chin-Ann J. Ong

**Affiliations:** ^1^ Department of Sarcoma, Peritoneal and Rare Tumours (SPRinT), Division of Surgery and Surgical Oncology, National Cancer Centre Singapore, Singapore, Singapore; ^2^ Department of Sarcoma, Peritoneal and Rare Tumours (SPRinT), Division of Surgery and Surgical Oncology, Singapore General Hospital, Singapore, Singapore; ^3^ Laboratory of Applied Human Genetics, Division of Medical Sciences, National Cancer Centre Singapore, Singapore, Singapore; ^4^ Department of Head and Neck Surgery, Division of Surgery and Surgical Oncology, National Cancer Centre Singapore, Singapore, Singapore; ^5^ SingHealth Duke-NUS Institute of Precision Medicine, National Heart Centre Singapore, Singapore, Singapore; ^6^ Cancer and Stem Biology Program, Duke-NUS Medical School, Singapore, Singapore; ^7^ Laboratory of Cancer Epigenome, Division of Medical Sciences, National Cancer Centre Singapore, Singapore, Singapore; ^8^ Lymphoma Genomics Translational Laboratory, Division of Medical Sciences, National Cancer Centre Singapore, Singapore, Singapore; ^9^ Department of Anatomical Pathology, Singapore General Hospital, Singapore, Singapore; ^10^ Pathology Academic Clinical Program, SingHealth Duke-NUS Academic Medical Centre, Singapore, Singapore; ^11^ Institute of Molecular and Cell Biology, ASTAR Research Entities, Singapore, Singapore; ^12^ SingHealth Duke-NUS Oncology Academic Clinical Program, Duke-NUS Medical School, Singapore, Singapore; ^13^ SingHealth Duke-NUS Surgery Academic Clinical Program, Duke-NUS Medical School, Singapore, Singapore; ^14^ Cancer Therapeutics Research Laboratory, Division of Medical Sciences, National Cancer Centre Singapore, Singapore, Singapore

**Keywords:** field cancerization, field change, synchronous head and neck cancers, transcriptomic, genomic

## Abstract

**Introduction:**

Field cancerization is suggested to arise from imbalanced differentiation in individual basal progenitor cells leading to clonal expansion of mutant cells that eventually replace the epithelium, although without evidence.

**Methods:**

We performed deep sequencing analyses to characterize the genomic and transcriptomic landscapes of field change in two patients with synchronous aerodigestive tract tumors.

**Results:**

Our data support the emergence of numerous genetic alterations in cancer-associated genes but refutes the hypothesis that founder mutation(s) underpin this phenomenon. Mutational signature analysis identified defective homologous recombination as a common underlying mutational process unique to synchronous tumors.

**Discussion:**

Our analyses suggest a common etiologic factor defined by mutational signatures and/or transcriptomic convergence, which could provide a therapeutic opportunity.

## 1 Introduction

The concept of field cancerization was first proposed by Slaughter et al. in 1953 (1) who posited that oral squamous cell carcinomas arise from a background of histologically normal but functionally abnormal tissues which develop a high incidence of multi-centric tumors. Molecular biomarker studies subsequently centered on deciphering whether field cancerization is attributed to polyclonal or monoclonal expansion of abnormal cells within the cancerized field (2–4). Recent publications utilizing lineage tracing techniques in murine models suggest that field change arises from imbalanced differentiation of individual basal progenitor cells leading to clonal expansion of mutant cells that eventually replace the entire epithelium (5). However, this murine model has not been validated in clinical scenarios of human field change. We were presented with a unique opportunity to study field cancerization in both dimensions of vertical (basal to squamous surface layers) and lateral (radial) spatial cellular genomic clonality in two patients who developed multiple synchronous squamous cell carcinomas (SCC) in the upper aerodigestive tract and esophagus. Both underwent total pharyngo-laryngo-esophagectomy (TPLE), providing valuable tumor and adjacent histologically normal tissue samples for integrative genomic and transcriptomic analysis, with the goal of providing insights into clinical field cancerization, novel therapeutic options, and comprehensive surveillance.

## 2 Case reports

### 2.1 Patient 1 (HN129)

Mr P was a 38-year-old smoker (10 pack-years) diagnosed with three synchronous SCCs involving the left pyriform sinus, post cricoid space, and lower esophagus. No distant metastases were detected radiologically. He underwent TPLE with gastric pull-up and bilateral neck dissection. Final histological diagnosis was pT2 moderately differentiated SCC of the hypopharynx, left pyriform sinus, and lower esophagus, with a single cervical metastasis (N1). The three tumors were histologically distinct. There was an absence of grossly malignant change spreading in the submucosa. Multiple discontinuous foci of severe squamous dysplasia were noted between tumors.

### 2.2 Patient 2 (HN146)

Ms S was a 50-year-old female smoker (20 pack-years) diagnosed with two synchronous SCCs involving the right pyriform sinus and upper esophagus. She underwent TPLE with gastric pull-up and bilateral neck dissection. Final histological diagnosis was pT1N0 SCC of the hypopharynx and upper esophagus. The tumors were distinct with histologically normal intervening mucosa and occasional small foci of low-grade dysplasia in the pharyngeal and esophageal mucosa.

### 2.3 Patient 3 (HN49)

Mr C was a 60-year-old male smoker (10 pack-years) and alcoholic. He developed metachronous supraglottic SCC 3 years after subtotal glossectomy with neck dissection for SCC of the tongue. Surgical samples enabled integrative analysis of synchronous tumors and matched adjacent normal mucosa.

## 3 Materials and methods

### 3.1 Patient selection and multi-sampling of specimens

We interrogated the mutational landscape of samples taken from three patients. Systematic multi-sampling of tumors and the intervening normal mucosa was conducted on tumors which were surgically removed as part of standard of care. Meticulous annotations of the specimen collection sites were recorded (**Supplementary Tables S1**, **S2**). This study was approved by the SingHealth Centralized Institutional Review Boards (CIRB 2008/467/B) and all patients gave individual informed consent.

Tissue samples were either snap frozen in liquid nitrogen immediately after collection and stored at −80°C or fixed in formalin and embedded in paraffin according to the usual clinical protocol. Blood from the eligible patients was drawn in two EDTA tubes and aliquoted into multiple cryotubes to be stored at −80°C.

### 3.2 DNA and RNA isolation for high throughput sequencing

DNA and RNA of the human tissue biopsies were extracted using the AllPrep DNA/RNA Mini Kit (QIAgen, Hilden, Germany, catalogue no. 80204), according to the manufacturer’s instructions. DNA bloods were extracted using QIAamp DNA blood mini kit (QIAgen, Hilden, Germany, catalogue no. 51104). For all sample types, the DNA and RNA were quantified using the Qubit dsDNA BR and RNA BR Assay kit (Life Technologies, Carlsbad, CA, USA, catalogue no. Q32850 and Q10211).

For the fresh frozen samples, whole-exome sequencing libraries were prepared using the Sureselect XT target enrichment kit (Agilent, Santa Clara, CA, USA; cat. #5500–0105, version 1.6). Libraries were constructed with an insert length of 300bp. Enriched libraries were sequenced on the Illumina Nextseq System (Illumina, San Diego, CA, USA) using the paired-end 150 bases configuration.

The RNA extracted from the same set of frozen samples was subjected to RNA sequencing. Enriched libraries were generated using the Truseq Stranded Total RNA Library Prep Gold kit (Illumina, San Diego, CA, USA; cat. #20020598) and subsequently sequenced on the HiSeq2000 system (Illumina) using the paired-end 76 bases run setting.

### 3.3 Gene set enrichment analysis

RNAseq data was analyzed using the DNAnexus platform (DNAnecus, Mountain View, CA, USA). Reads were mapped using TopHat2 v2·0·12 and CuffDiff v2·1·1 on the genome version ucsc_hg19. Fragments per Kilobase of transcript per Million (FPKM) and fragment counts for each transcript, primary transcript and gene in each sample was normalized as part of CuffDiff.

### 3.4 TMA construction, and immunohistochemistry staining and scoring

Tissue microarrays (TMAs) were constructed using tumors collected from 328 patients treated for histologically confirmed HNSCC and who underwent treatment at the National Cancer Centre Singapore or Singapore General Hospital between 1 998 and 2010. This study was approved by the SingHealth Centralized Institutional Review Boards (CIRB 2011/678/B and 2007/438/B). TMA blocks were sectioned into 4µM slices and mounted on slides. IHC staining was performed using the BOND-MAX autostainer (Leica Microsystems, Ltd; Milton Keynes, UK) according to the manufacturer’s recommendations.

IHC staining were assessed by two independent scorers blinded to any prior information of clinicopathological variables and survival data. Scoring was performed by assigning the intensity of the stain to a value of 0 (no positive staining) to 3 (strong positive staining). Results were binarized to low (0,1) or high (2,3) expression of marker.

### 3.5 Laser capture microdissection and targeted resequencing

Using the Shh image with pathological annotations as a reference, cutting outlines were drawn closely around individual cells to prevent tissue contamination. Pathologist identified basal, squamous, and tumor areas for microdissection (**Supplementary Figure S1**). Formalin-fixed paraffin-embedded (FFPE) samples matched to the sequenced frozen specimens were identified to be subjected to LCM and targeted resequencing. Briefly, 5µm tissue sections were mounted on the PEN membrane slides (Arcturus, Life Technologies). The tissue section was deparaffined with xylene, dehydrated in a series of graded ethanol and stained with 3:1 hematoxylin:eosin (Merck, Rahway, NJ, USA, catalogue no. 1.05174 and Sigma-Aldrich, St. Louis, MO, USA, catalogue no. HT110116). Laser-captured cells were collected on CapSure™ Macro LCM caps (Thermo Fisher Scientific, Waltham, MA, USA, catalogue no. LCM0211) using the Arcturus XT LCM instrument (Life Technologies). Microdissected samples were stored at −80°C until further processing.

### 3.6 Targeted resequencing

DNA isolation from the microdissected samples were carried out using the QIAamp DNA FFPE tissue kit (QIAgen, catalogue no. 56404). We selected 151 genes associated with head and neck cancer for target enrichment (**Supplementary Tables S3**). The gene panel consisted of genes derived from our in-house exome sequencing data and the scholarly literatures (6, 7). The DNA libraries were constructed using the SureSelectXT HS Target Enrichment System (Agilent Technologies, Santa Clara, CA, USA). Sequencing was performed on the HiSeq 4000 system (Illumina) using the paired-end 150 bases run.

### 3.7 Analysis of genomic data and copy number changes

Raw sequencing reads generated through whole-exome sequencing were aligned to the human reference genome (hs37d5) using BWA-MEM (8), and then post-processed to sort the reads by genomic coordinate and to remove PCR duplicates using Sambamba (9). The resulting alignment files in the BAM format were used for the subsequent somatic mutation calling step. Briefly, the Strelka2 program was used to identify somatic mutations in tissue (either tumor or adjacent mucosa), with patient whole-blood used as a control (10). Candidate single nucleotide variants (SNVs) that passed default Strelka2 filtering criteria, and that had a variant allele frequency (VAF) exceeding 20% were retained. These variants were then annotated using Annovar to determine their impact on protein sequence, frequency in population variation databases as well clinical variant databases, and *in-silico* functional prediction scores (11). The limma R package was used to read CGH data, perform background correction and normalize within arrays. The snapCGH R package was then used for genome segmentation, genes were classified as gained (≥3 copies), lost (≤1 copy) or normal based on log2 ratios. Genes were compared for copy number status across samples.

### 3.8 Mutational signature analysis

The *deconstructSigs* R package was used to estimate the contribution of known mutational processes to the somatic mutations observed in a tumor or adjacent mucosa sample (12, 13). The known mutational processes were obtained from the COSMIC mutational signatures database (https://cancer.sanger.ac.uk/cosmic/signatures). The algorithm produces a list of mutational processes predicted to be present in a sample and their relative proportions.

### 3.9 Analysis of targeted resequencing data

Targeted regions were sequenced to an average coverage of 1503X. The somatic mutation calling and variant annotation process was identical to the one used for WES data above. Variants were filtered to retain only SNVs that were nonsense, splicing and nonsynonymous. Only variants with a VAF exceeding 10% in at least one sample were considered. Variants that had SIFT (14) and Polyphen2 (15) HDIV predictions were required to have damaging (SIFT) and damaging or probably damaging (Polyphen2 HDIV) predictions, respectively.

## 4 Results

### 4.1 Genomic divergence is greater in synchronous than in metachronous tumors

**Figure 1:**
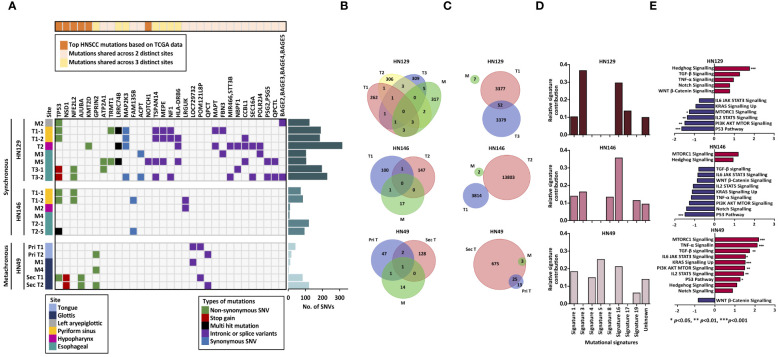
Synchronous tumors of the aerodigestive tract possess distinct tumor biology from that of metachronous tumors. **(A)** Heatmap illustrating the mutational landscape of the samples derived from three distinct patients. Frequency of single nucleotide variants in tumor and adjacent normal mucosa indicate lower mutation counts in normal mucosa (M) compared to that of tumor (T) in all three patients. **(B)** No shared mutations were identified between the normal mucosa and synchronous tumors while merely one shared mutation was identified in metachronous tumors and mucosa. **(C)** Few copy number alterations are shared across the samples in both synchronous and metachronous tumors. **(D)** Signature 3 was common to synchronous tumors whereas Signature 4 was unique to metachronous tumors. **(E)** Differential modulation of signaling pathways is seen between synchronous (HN129) and metachronous (HN49) tumors via gene set enrichment analysis (GSEA). Normalized expression values are reflected in the bar charts, where *p*-value < 0.05 is represented with blue (down-regulated) or red (up-regulated). Our data revealed that hedgehog pathway was significantly enriched in synchronous tumors. SNV, single nucleotide variants; M, mucosa; T, tumor; Shh, sonic hedgehog.

Genomic alterations in synchronous and metachronous tumors were determined by whole-exome sequencing (WES) and array comparative genomic hybridization (aCGH) on bulk samples of paired tumor and adjacent normal mucosa from each of the three patients (**Supplementary Tables S1**, **S2**). WES results demonstrated consistently lower overall number of SNV in adjacent normal mucosa compared to tumors in all three patients (**Figures 1A, B**). Strikingly, normal mucosa adjacent to HN146 (synchronous) and HN49 (metachronous) tumors were almost completely mutation-free, while adjacent normal mucosa in the remaining patient (HN129) had a median (range) of 105 (7%) SNVs. The median (range) SNV counts of synchronous tumors (HN129 and HN146) were 197 (14%) and 91 (23%), respectively, while that of the metachronous tumor (HN49) was lower at 63 (22%) (**Figure 1A**). Analyses of copy number variants (CNV) showed similar results with low copy number alterations ranging from 3–7 CNVs in normal mucosa across all three patients examined. The mean CNVs in synchronous tumors (HN129 and HN146) were 3378 and 8809 respectively, whereas that in metachronous tumor (HN49) was comparably lower at 344 (**Figure 1C**). Changes in the status of MYC copy number were observed in both synchronous and metachronous tumors whereas all the blood and most normal mucosa, except for HN129 M2, have normal MYC copy number. Among the samples with MYC copy number aberrations, only one synchronous tumor had MYC copy number amplification while MYC copy number gains were noted in the remaining synchronous and metachronous samples. The heterogeneity in the copy number changes suggests that synchronous tumors are unlikely driven by somatic copy number alternation burden (**Supplementary Tables S4**).

Conventional field change theory suggests that tumors growing in a cancerized field start from a clonal outgrowth of a single malignant cell that shares tumorigenic ‘founder’ mutations with the adjacent ‘normal’ tissue (16). Our analysis of shared genetic alterations within matched tumor-normal combinations of patients with synchronous tumors (HN129 and HN246) showed no shared mutations among the different synchronous tumors of each patient (**Figure 1B**), while only one shared mutation was identified in the metachronous tumors. Additionally, there were no shared CNVs among synchronous tumors or the cognate matched intervening mucosa (**Figure 1C**). These results refute the hypothesis of founder mutations as an early event in synchronous field cancerization.

### 4.2 Mutational signatures defining early events in field cancerization

There is widespread interest in the mutagenic processes that drive carcinogenesis from known and unknown etiological factors. These processes are defined by their mutational signatures (12). Given the paucity of common genomic aberrations, we postulated that mutagenic signatures could define field change, and more specifically differentiate synchronous from metachronous tumors. *De novo* mutational signature analysis (12, 17) revealed common trends among the three patients, such as the prevalence of Signatures 1 and 16 (**Figure 1D**). Signature 1 is a mutational signature found in most cancer subtypes and is associated with small insertions and deletions driven by spontaneous deamination of 5-methylcytosine. Signature 16 is of unknown etiology and is characterized by distinct transcriptional strand bias for T>C mutations occurring in the transcribed strand (18, 19).

Interestingly, we noted that Signature 4 (usually attributable to smoking) was prevalent in HN49, but not in the two synchronous patients. In contrast, Signature 3 dominated in both patients with synchronous tumors. This is intriguing as Signature 3 is often associated with failure of DNA double-strand break repair by homologous recombination (18). In cancers such as breast and pancreatic cancers, Signature 3 is strongly associated with germline or somatic BRCA1/BRCA2 mutations, which are well-established indicators for susceptibility to PARP inhibitors (20, 21). Re-examination of genomic data for these two patients did not reveal any germline or somatic BRCA mutations. Even so, the concept of using PARP inhibitors to treat HNSCC patients with tumors that exhibit mutational Signature 3 may still be plausible. Studies have suggested the potential of using Signature 3 as a decision support to select patients who may benefit from platinum-based therapy or PARP inhibitors, even in the absence of BRCA1 or BRCA2 mutations (22).

### 4.3 Paradoxical genomic divergence with transcriptomic convergence in synchronous tumors

Transcriptomic data support the notion that the concept of field cancerization relates more to etiologies that alter the microenvironment and drive mutagenic events during carcinogenesis, rather than actual initiating founder mutations (23). Lineage tracing experiments performed in a controlled diethylnitrosamine (DEN)/sorafenib carcinogenesis mouse model showed that high grade dysplasias (HGD) share highly similar transcriptomic profiles despite being of polyclonal origin (24). These data support the possibility that transcriptomic changes pre-date genomic alterations, and reflect cellular phenotypic responses to the initial mutagenic insult. Moreover, this ‘transcriptomic convergence’ may provide a clue to the ‘cell of origin’ that drives field cancerization, and unravel therapeutic opportunities that prevail across genetically divergent tumors.

We therefore subjected matching sets of adjacent mucosa and tumor samples of all three patients to RNA sequencing. Gene set enrichment analysis (GSEA) of RNA-seq data revealed that synchronous and metachronous tumors were driven by distinct biological processes. Hedgehog (Hh) pathway was identified as the key signaling pathway in synchronous cancers (normalized enrichment score (NES)=1·77, *p*-value=0·002) but was not significantly upregulated in metachronous tumors (NES=1·09, *p*-value=0·324) (**Figure 1E**). Differential gene expression analysis of the samples studied in GSEA showed concordance with the GSEA data where targets involved in the Hedgehog pathway were more enriched in the synchronous tumors compared to the metachronous tumors when normalized to their respective normal tissues (**Supplementary Figure S2**). To validate this observation, we assessed the protein expression level of Sonic Hedgehog (SHH), which is a key regulator within the Hh signaling pathway, in FFPE tumor samples harvested from the same corresponding site as the frozen tumors, which were subjected to genomic and transcriptomic profiling across the three case studies. Immunohistochemical (IHC) staining of SHH markers in these tumors demonstrated higher staining intensity across all the synchronous tumors compared to the metachronous tumors. (**Supplementary Figure S3**). More importantly, to understand the clinical relevance of Hh upregulation in head and neck cancers, we performed IHC staining on a cohort of head and neck SCC (HNSCC) patients (n=328) (**Supplementary Figure S4A**). Approximately a quarter of all patients demonstrated upregulated Hh signaling, which was associated with poor prognosis (*p*=0·014) (**Supplementary Figure S4B**). We validated our observation in an independent cohort of HNSCC patients in The Cancer Genome Atlas (TCGA) database (6), in whom high expression of SHH was significantly associated with shorter overall survival (*p*=0·015) (**Supplementary Figure S4C**).

### 4.4 Hedgehog signaling in basal cells as putative transcriptomic driver of synchronous tumors

**Figure 2:**
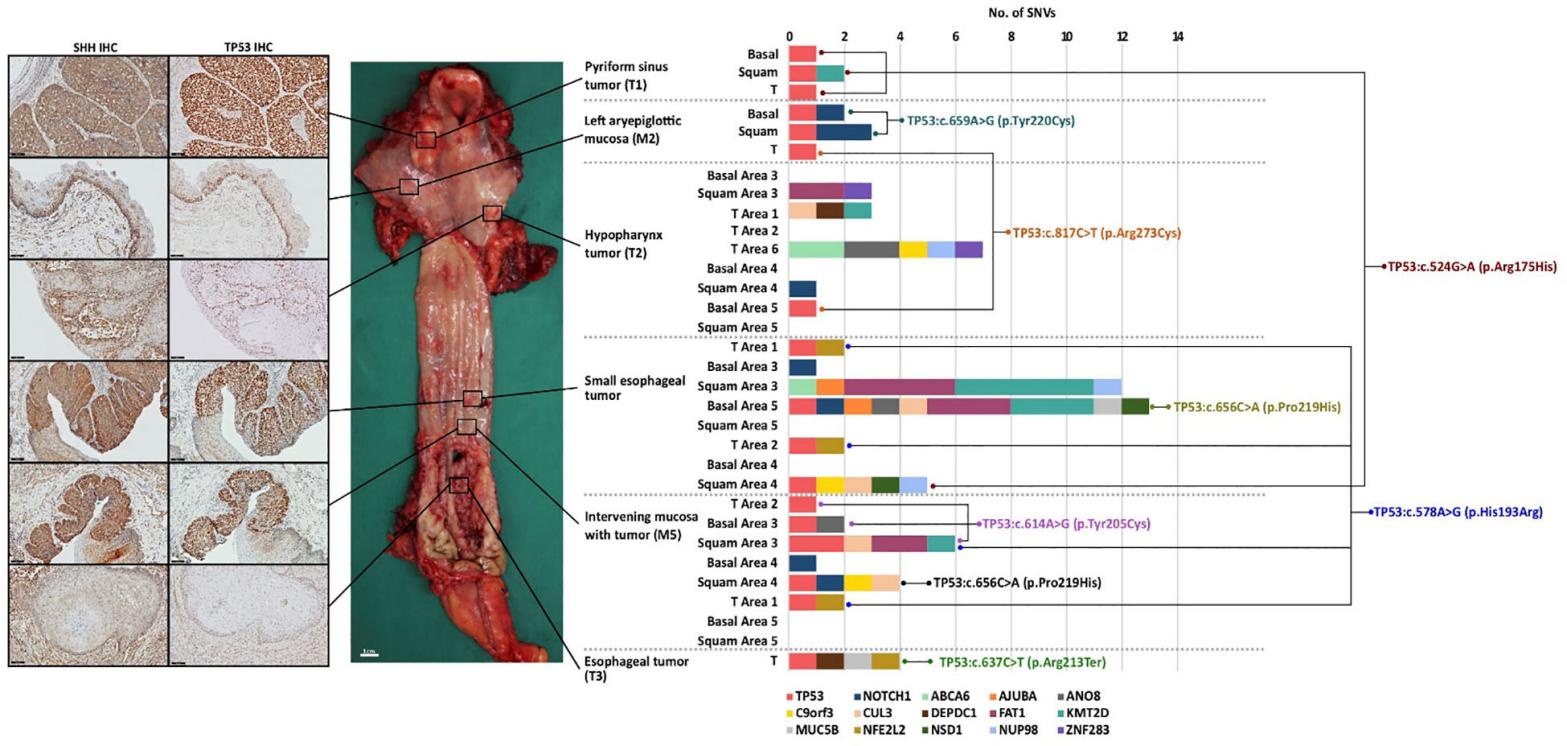
Synchronous tumors of the aerodigestive tract display a unique profile of genomic divergence and transcriptomic convergence. The TP53 transcript ENST00000269305.4 was used as reference for the identification of SNVs in TP53. SNV, single nucleotide variants; M, mucosa; T, tumor; Shh, sonic hedgehog.

**Figure 3:**
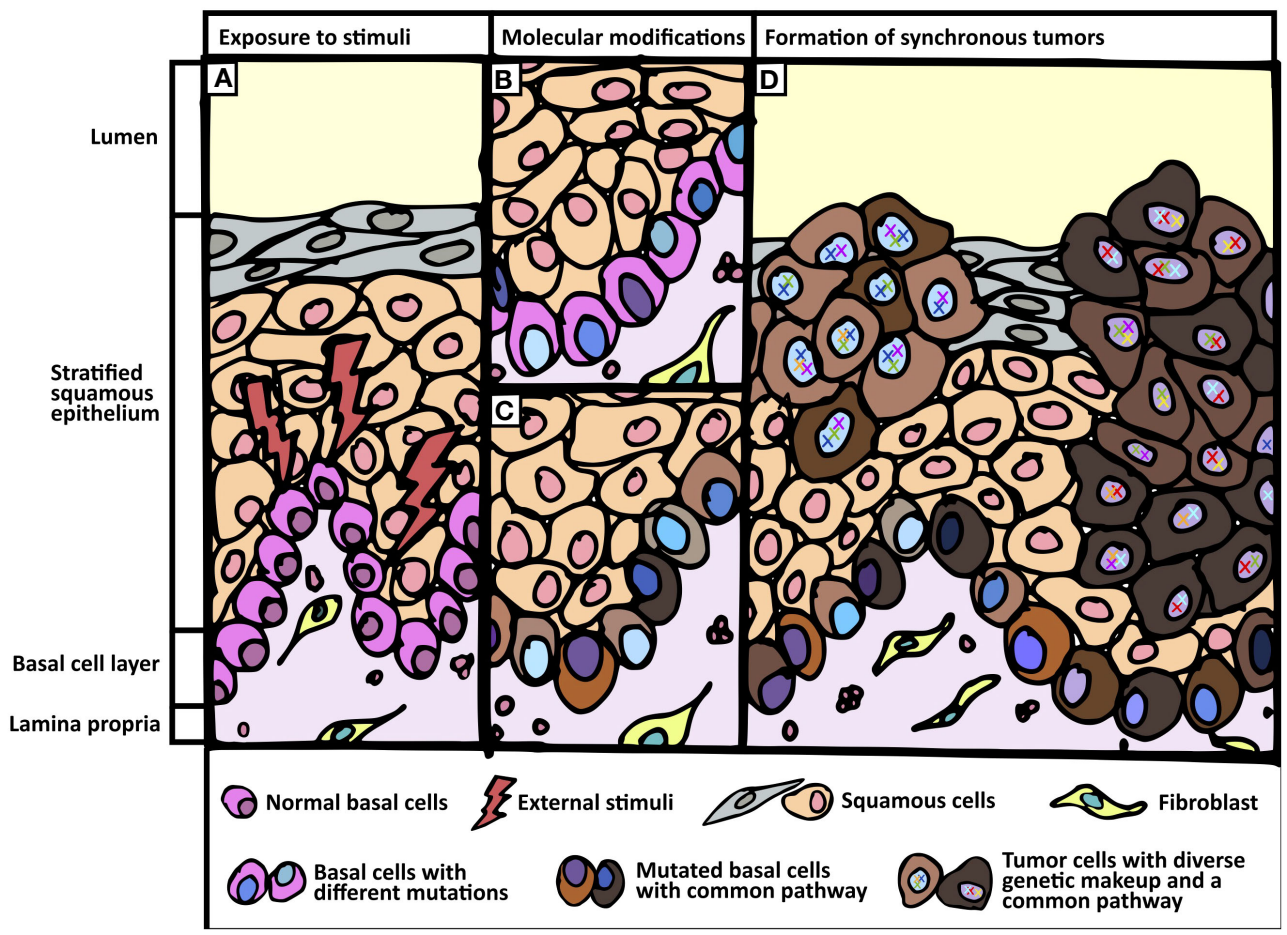
Proposed field change phenomenon. **(A)** Exposure of normal tissue to external stimuli. **(B, C)** Basal cell layer undergo genetic and transcriptomic modifications when exposed to external stimuli. **(B)** Different shades of blue in the nuclei of basal cells represent genomic divergence. **(C)** Varying shades of brown in the cytoplasm of basal cells represent common transcriptomic profile (e.g. Hedgehog pathway). **(D)** Formation of synchronous tumors harboring common signaling pathway but divergent genomic make-up.

Our IHC data demonstrated that in many tumors, Hh upregulation was prevalent in both tumor cells and basal cell layers of adjacent normal mucosa. This was striking across the entire mucosa (tumor, normal and dysplastic) of the two patients with synchronous tumors. Hh signaling is known to affect multiple aspects of DNA repair in cells (25), including inhibition of p53 possibly via activation of Mdm2 (26, 27), hence driving chromosomal instability. Few studies have demonstrated links between TP53 inactivation and activated Hh signaling (28–30) in promoting cancer development. Due to the complexity of this association, the underlying mechanism remains unclear and thus future work is required to further elucidate this. In this study, we posited that transcriptional activation of the Hh pathway may be the first step to p53 inactivation, followed by a second hit where other genes, including p53, are mutated. IHC confirmed the former, where p53 accumulation was present across the entire mucosa in both cases, in concert with Hh overexpression. This was particularly apparent in the basal layer across histologically normal and dysplastic mucosa. To determine the distribution of subsequent genetic events, we performed laser capture microdissection of normal and dysplastic mucosa, as well as of adjacent synchronous tumors in HN129. DNA was extracted from tumor, basal and squamous compartments and subjected to targeted sequencing using a panel of mutations detected by WES in HN129 and other known oncogenic drivers of HNSCC (**Supplementary Tables S3**, **Supplementary Figure S1**). The most striking finding was the distribution pattern of different SNVs in TP53 (ENST00000269305.4) across basal, squamous and tumor cells microdissected from multiple sites of the aerodigestive tract (**Figure 2**). In ‘normal’ mucosa immediately adjacent to tumors, we found specific TP53 mutations shared between the basal and tumor areas, or basal and squamous layers. However, in other situations, TP53 mutations were discordant between tumor and adjacent basal layer. We observed that a specific TP53 mutation, TP53:c.524G>A (p.Arg175His), co-occurred across two geographical distinct samples, namely the microdissected tumor component of pyriform sinus tumor (T1) and squamous area of small esophagus tumor (T3). Intriguingly, we also noted that two unique TP53 mutations, namely TP53:c.614A>G (p.Tyr205Cys) and TP53:c.578A>G (p.His193Arg), were present in the same microdissected tissue, squamous area 3 of the intervening mucosa with tumor (M5), suggesting that clonal expansion was not present in our samples. Importantly, variant allele frequencies were significantly higher in tumors compared to histologically normal or dysplastic regions. Furthermore, the basal layer appeared to have fewer SNVs (apart from TP53) compared to squamous layers or tumors. This suggests that TP53 mutations are early events in the basal layer, which gain subsequent alterations that drive it either to terminal differentiation in the squamous layer or tumor formation (**Figure 3**). Contrastingly, certain late genetic events are only seen in tumors, and never in the basal or squamous layers (e.g. NFE2L2) (**Figures 1A**, **2**).

## 5 Discussion

Through deep sequencing analyses, we profiled in-depth genomic and transcriptomic landscapes of field change in two patients with synchronous aerodigestive tract tumors. Our data support the emergence of numerous genetic alterations in cancer-associated genes, likely from the basal layer, but refutes the hypothesis that either a single or a limited panel of founder mutations underpin this phenomenon. Instead, our analyses suggest a common etiologic factor defined by mutational signatures and/or transcriptomic convergence. Targeted resequencing of subcellular components demonstrated distinct mutational profiles of squamous epithelium and tumors coupled with a paucity of mutations in the basal cell layer, suggesting that the latter likely contributes the cell of origin for synchronous tumors.

Activation of Hh pathway, specifically in the basal layer, appears to be an early event that defines field change in this context. This could allow a permissive environment by disrupting p53 activity, followed by low-level mutations in critical genes such as TP53 as a putative second hit. Subsequent genetic events accumulate and develop into distinct areas of dysplasia and tumors, each genetically distinct. Clonal origin supporting classical field cancerization theory does not seem to occur in this context. Contrarily, etiologic field change is supported by convergent transcriptomes with independent mutations generating polyclonal tumors.

While this study describes a small case series of three patients with SCC, the systematic approach of combining molecular pathology and multi-omics data allows for meaningful analyses that provide early observations suggesting a different model for the classical field cancerization theory and requires further validation in future studies. These findings have important implications in cancer biology but also in clinical oncology, especially extending beyond squamous cell cancers. Identification of transcriptomic convergence could provide a therapeutic opportunity that cuts across all tumors in a field, regardless of genetic diversity; here, we posit that targeting the Hh pathway is a novel option targeting the tumor and basal cells, where these originate. Similarly, despite the diversity of mutations across synchronous tumors, understanding mutational signatures may provide an interventional strategy. In our context, the mutational profiles of our case series demonstrated the fact that there were mutational signatures which were unique to the synchronous tumors and metachronous tumors. While all the three patients within our case series had history of tobacco smoking, Signature 4, associated with smoking, was only prevalent in metachronous tumor (HN49), but not in the two synchronous patients. Intriguingly, overrepresentation of Signature 3, as found common to the synchronous tumors in our case series, normally ascribed to “BRCA-ness” leads to speculation that these tumors may be responsive to PARP-inhibitors. This is further supported by our transcriptomic data where PARP targets, such as PARP1 and LIG3, were more enriched in the synchronous tumors compared to the metachronous tumors (31) (**Supplementary Figure S5**). Both therapeutic strategies may have important roles in treatment or secondary prevention. Alternatively, routine screening procedures such as sampling of the esophagus via the Cytosponge could help to document genomic divergence and incorporate testing for Shh signaling pathway activation, thereby capturing clinically relevant oncogenic factors beyond those imputed by classical theory (32).

## Data availability statement

The datasets used and/or analyzed during the current study are available from the corresponding author on reasonable request.

## Ethics statement

The studies involving humans were approved by SingHealth Centralized Institutional Review Boards. The studies were conducted in accordance with the local legislation and institutional requirements. The participants provided their written informed consent to participate in this study.

## Author contributions

QXT: Data curation, Formal analysis, Investigation, Validation, Visualization, Writing – original draft, Writing – review & editing. NBS: Formal analysis, Investigation, Validation, Writing – review & editing. WKL: Formal analysis, Investigation, Validation, Writing – review & editing. DRYY: Data curation, Formal analysis, Investigation, Validation, Writing – original draft, Writing – review & editing. SML: Data curation, Formal analysis, Investigation, Validation, Writing – review & editing. JWST: Formal analysis, Investigation, Validation, Writing – review & editing. SJJT: Data curation, Formal analysis, Investigation, Validation, Writing – review & editing. JH: Formal analysis, Investigation, Validation, Writing – review & editing. YL: Formal analysis, Investigation, Validation, Writing – review & editing. GN: Formal analysis, Investigation, Validation, Writing – review & editing. CYLC: Formal analysis, Investigation, Validation, Writing – review & editing. WG: Formal analysis, Investigation, Validation, Writing – review & editing. KKNK: Formal analysis, Investigation, Validation, Writing – review & editing. CCYN: Formal analysis, Investigation, Validation, Writing – review & editing. VR: Formal analysis, Investigation, Validation, Writing – review & editing. JSMW: Formal analysis, Validation, Writing – review & editing. CJS: Formal analysis, Validation, Writing – review & editing. CKO: Formal analysis, Investigation, Validation, Writing – review & editing. TKHL: Formal analysis, Investigation, Validation, Writing – review & editing. BTT: Supervision, Writing – original draft, Writing – review & editing. OLK: Writing – review & editing, Supervision, Writing – original draft. CSC: Supervision, Writing – original draft, Writing – review & editing. KCS: Supervision, Writing – original draft, Writing – review & editing. NGI: Conceptualization, Funding acquisition, Methodology, Project administration, Resources, Supervision, Visualization, Writing – original draft, Writing – review & editing. C-AJO: Conceptualization, Funding acquisition, Methodology, Project administration, Resources, Supervision, Visualization, Writing – original draft, Writing – review & editing. JXT: Formal analysis, Validation, Writing - review and editing.
