# Supplementary material for: Transcriptomic convergence despite genomic divergence drive field cancerization in synchronous squamous tumors
Source: Front Oncol. 2024 Jun 13;14:1272432. doi: 10.3389/fonc.2024.1272432 (PMC11208456; doi:10.3389/fonc.2024.1272432)
Supplement: Supplementary file 1 [file DataSheet_1.docx]

Supplementary Material

# Supplementary Table

| Supplementary Table S1. List of tissue samples subjected to exome sequencing and RNA sequencing. | |
| --- | --- |
| Patient | **Sample ID** |
| HN129 | Pyriform sinus tumor (T1-1) |
|  | Pyriform sinus tumor (T1-2) |
|  | Hypopharynx tumor (T2-1) |
|  | Esophageal tumor (T3-1) |
|  | Esophageal tumor (T3-2) |
|  | Left aryepiglottic fold mucosa (M2) |
|  | Middle esophageal mucosa (M3) |
|  | Intervening mucosa between T1 & T3 (M5) |
| HN146 | Pyriform sinus tumor (T1-1) |
|  | Pyriform sinus tumor (T1-2) |
|  | Esophageal tumor (T2-1) |
|  | Esophageal tumor (T2-5) |
|  | Hypopharyngeal mucosa (M2) |
|  | Middle esophageal mucosa (M4) |
| HN49 | Primary tongue tumor (T1) |
|  | Primary tongue tumor (T2) |
|  | Secondary glottis tumor (T1) |
|  | Secondary glottis tumor (T2) |
|  | Glottis mucosa (M1) |
|  | Glottis mucosa (M4) |

| Supplementary Table S2. Summary table of samples used in exome sequencing and RNA-seq analysis |
| --- |

| Subject ID | Normal mucosa | Synchronous tumor | Metachronous tumor |
| --- | --- | --- | --- |
| HN129 | 3 | 5 | 0 |
| HN146 | 2 | 4 | 0 |
| HN49 | 2 | 0 | 4 |

| **Supplementary Table S3.** List of genes and mutation regions included in the targeted sequencing | | |
| --- | --- | --- |
| **SN** | **Gene** | **DNA position** |
| 1 | MIIP | 1:12082261-12082261,g>a |
| 2 | OR6N1 | 1:158735597-158735597,c>g |
| 3 | EIF4G3 | 1:21137288-21137288,c>t |
| 4 | GPR137B | 1:236341899-236341899,c>g |
| 5 | RYR2 | 1:237955578-237955578,c>a |
| 6 | OR6F1 | 1:247875551-247875551,a>c |
| 7 | KHDRBS1 | 1:32505121-32505121,c>g |
| 8 | ZBTB8B | 1:32937163-32937163,t>c |
| 9 | MPL | 1:43805144-43805144,g>c |
| 10 | DEPDC1 | 1:68955208-68955208,t>c |
| 11 | PDZD8 | 10:119078399-119078399,c>g |
| 12 | DOCK1 | 10:129055683-129055683,g>a |
| 13 | NRG3 | 10:84718743-84718743,c>t |
| 14 | RPP30 | 10:92663000-92663000,c>g |
| 15 | LGI1 | 10:95552964-95552964,t>g |
| 16 | GUCY1A2 | 11:106810503-106810503,c>g |
| 17 | HYLS1 | 11:125769499-125769499,c>t |
| 18 | MUC5B | 11:1267055-1267055,c>t |
| 19 | NUP98 | 11:3733830-3733830,c>g |
| 20 | TCN1 | 11:59631560-59631560,c>t |
| 21 | CATSPER1 | 11:65790511-65790511,c>t |
| 22 | MTMR2 | 11:95591751-95591751,t>c |
| 23 | UTP20 | 12:101748892-101748892,c>g |
| 24 | PRDM4 | 12:108136012-108136012,t>c |
| 25 | UBE3B | 12:109927867-109927867,g>c |
| 26 | CACNA1C | 12:2794921-2794921,g>a |
| 27 | CACNB3 | 12:49220583-49220583,g>a |
| 28 | DHH | 12:49487995-49487995,c>t |
| 29 | GRASP | 12:52404822-52404822,g>a |
| 30 | PMEL | 12:56351760-56351760,c>t |
| 31 | ITGBL1 | 13:102345033-102345033,g>c |
| 32 | USP12 | 13:27680026-27680026,g>a |
| 33 | FAM48A | 13:37605982-37605982,g>c |
| 34 | PCDH17 | 13:58207437-58207437,c>a |
| 35 | CLEC14A | 14:38724494-38724494,g>c |
| 36 | MDGA2 | 14:47343247-47343247,c>g |
| 37 | ZBTB1 | 14:64989282-64989282,g>a |
| 38 | ZBTB1 | 14:64989708-64989708,g>c |
| 39 | MLH3 | 14:75515175-75515175,t>c |
| 40 | LYSMD4 | 15:100271995-100271995,g>a |
| 41 | VPS18 | 15:41192647-41192647,a>g |
| 42 | MYO5A | 15:52718079-52718079,t>c |
| 43 | TLN2 | 15:63029280-63029280,g>a |
| 44 | CHRNA3 | 15:78894545-78894545,c>t |
| 45 | PARN | 16:14540826-14540826,c>t |
| 46 | NUBP2 | 16:1837701-1837701,g>c |
| 47 | RP11-231C14.2 | 16:29401282-29401282,c>g |
| 48 | CTCF | 16:67670725-67670725,g>a |
| 49 | ZC3H18 | 16:88664618-88664618,a>g |
| 50 | NOS2 | 17:26096592-26096592,a>g |
| 51 | SDF2 | 17:26976164-26976164,g>a |
| 52 | SUPT6H | 17:27024982-27024982,t>c |
| 53 | OR3A1 | 17:3195546-3195546,c>t |
| 54 | LYZL6 | 17:34261830-34261830,g>c |
| 55 | KLHL11 | 17:40011113-40011113,t>c |
| 56 | ACLY | 17:40042468-40042468,c>t |
| 57 | ABCA6 | 17:67079399-67079399,c>a |
| 58 | MXRA7 | 17:74684630-74684630,g>a |
| 59 | TP53 | 17:7578212-7578212,g>a |
| 60 | TP53 | 17:7578406-7578406,c>t |
| 61 | RNF213 | 17:78262531-78262531,c>g |
| 62 | ZSCAN30 | 18:32833895-32833895,c>t |
| 63 | CXXC1 | 18:47811521-47811521,g>a |
| 64 | PTPRM | 18:7888301-7888301,a>g |
| 65 | ANO8 | 19:17435541-17435541,c>t |
| 66 | TSHZ3 | 19:31769424-31769424,a>c |
| 67 | SIPA1L3 | 19:38682903-38682903,c>g |
| 68 | PHLDB3 | 19:44008099-44008099,c>g |
| 69 | ZNF283 | 19:44351834-44351834,c>t |
| 70 | PPP1R15A | 19:49377721-49377721,g>a |
| 71 | MZF1 | 19:59081870-59081870,c>t |
| 72 | MERTK | 2:112786316-112786316,g>a |
| 73 | GALNT13 | 2:155295236-155295236,g>a |
| 74 | GCG | 2:163000639-163000639,c>t |
| 75 | COL3A1 | 2:189873767-189873767,g>a |
| 76 | ITSN2 | 2:24471601-24471601,g>a |
| 77 | GCKR | 2:27730106-27730106,c>g |
| 78 | AHSA2 | 2:61413614-61413614,t>g |
| 79 | USP34 | 2:61622363-61622363,t>c |
| 80 | KDM3A | 2:86718115-86718115,c>t |
| 81 | RBM12 | 20:34241794-34241794,t>c |
| 82 | RBM39 | 20:34317250-34317250,c>g |
| 83 | PCIF1 | 20:44576312-44576312,c>t |
| 84 | NCOA3 | 20:46266456-46266456,a>g |
| 85 | THOC5 | 22:29924124-29924124,g>a |
| 86 | MKL1 | 22:40814896-40814896,c>t |
| 87 | BOC | 3:113005529-113005529,a>g |
| 88 | ASTE1 | 3:130743162-130743162,a>t |
| 89 | CPB1 | 3:148562328-148562328,g>a |
| 90 | CP | 3:148924097-148924097,c>t |
| 91 | P2RY1 | 3:152554181-152554181,g>a |
| 92 | SLITRK3 | 3:164906755-164906755,c>t |
| 93 | ATP11B | 3:182583395-182583395,c>g |
| 94 | FAM208A | 3:56667784-56667784,g>a |
| 95 | ADAMTS9 | 3:64527032-64527032,c>g |
| 96 | PRRT3 | 3:9990480-9990480,a>g |
| 97 | NFKB1 | 4:103518749-103518749,c>t |
| 98 | TMEM184C | 4:148550778-148550778,g>a |
| 99 | FSTL5 | 4:162307302-162307302,c>a |
| 100 | SPATA18 | 4:52927035-52927035,c>t |
| 101 | SLC4A4 | 4:72215737-72215737,g>t |
| 102 | AFF1 | 4:88026787-88026787,g>a |
| 103 | FBN2 | 5:127800458-127800458,c>t |
| 104 | SLC26A2 | 5:149357250-149357250,c>t |
| 105 | CDH10 | 5:24593540-24593540,g>t |
| 106 | RPS12 | 6:133138635-133138635,c>g |
| 107 | CCHCR1 | 6:31124517-31124517,c>t |
| 108 | TAF8 | 6:42044867-42044867,c>t |
| 109 | KLC4 | 6:43041705-43041705,t>a |
| 110 | GABRR1 | 6:89895154-89895154,t>c |
| 111 | THSD7A | 7:11446595-11446595,c>t |
| 112 | TRIM24 | 7:138210005-138210005,g>c |
| 113 | PARP12 | 7:139727080-139727080,g>a |
| 114 | DGKB | 7:14789779-14789779,c>g |
| 115 | HECW1 | 7:43490522-43490522,c>a |
| 116 | KDELR2 | 7:6502625-6502625,t>c |
| 117 | TYW1 | 7:66483095-66483095,c>g |
| 118 | PCLO | 7:82579541-82579541,g>a |
| 119 | RIMS2 | 8:105026790-105026790,g>a |
| 120 | DCSTAMP | 8:105367133-105367133,a>c |
| 121 | ADAM9 | 8:38879163-38879163,a>g |
| 122 | SNTG1 | 8:51415428-51415428,g>t |
| 123 | RALYL | 8:85799900-85799900,g>t |
| 124 | RUNX1T1 | 8:93074908-93074908,c>t |
| 125 | NR4A3 | 9:102595023-102595023,c>a |
| 126 | CYLC2 | 9:105765522-105765522,c>t |
| 127 | EHMT1 | 9:140707459-140707459,c>g |
| 128 | PCSK5 | 9:78969022-78969022,c>t |
| 129 | C9orf3 | 9:97535334-97535334,c>t |
| 130 | NRK | X:105193718-105193718,c>a |
| 131 | GPR112 | X:135494484-135494484,t>a |
| 132 | NLGN4Y | Y:16942344-16942344,g>a |
| 133 | CDKN2A | Coding regions |
| 134 | FAT1 | Coding regions |
| 135 | TP53 | Coding regions |
| 136 | CASP8 | Coding regions |
| 137 | AJUBA | Coding regions |
| 138 | PIK3CA | Coding regions |
| 139 | NOTCH1 | Coding regions |
| 140 | KMT2D | Coding regions |
| 141 | NSD1 | Coding regions |
| 142 | HLA-A | Coding regions |
| 143 | TGFBR2 | Coding regions |
| 144 | HRAS | Coding regions |
| 145 | FBXW7 | Coding regions |
| 146 | RB1 | Coding regions |
| 147 | PIK3R1 | Coding regions |
| 148 | TRAF3 | Coding regions |
| 149 | NFE2L2 | Coding regions |
| 150 | CUL3 | Coding regions |
| 151 | PTEN | Coding regions |

**Supplementary Table S4.** Copy number analysis of MYC across all synchronous and metachronous samples.

| **HN129** | | | | | | | | | | |
| --- | --- | --- | --- | --- | --- | --- | --- | --- | --- | --- |
|  | **Blood** | **T1-1** | **T1-2** | **T2-1** | **T3-1** | **T3-2** | **M2** | **M3** | **M5** |  |
| **MYC** | 0.015742 | -0.07782 | 0.027923 | 0.277531 | -0.14516 | -0.03005 | 0.291521 | 0.212328 | -0.02192 |  |
| **COPYRATIO** | 1.010971 | 0.947487 | 1.019543 | 1.212119 | 0.90428 | 0.979384 | 1.22393 | 1.158556 | 0.984924 |  |

| **HN146** | | | | | | | | |
| --- | --- | --- | --- | --- | --- | --- | --- | --- |
|  | **Blood** | **T1-2** | **T1-1** | **T2-1** | **T2-5** | **M2** | **M4** | **M5** |
| **MYC** | 0.049238 | 0.42662 | 0.160797 | 1.041563 | 0.680835 | -0.06416 | -0.16104 | -0.04299 |
| **COPYRATIO** | 1.034718 | 1.344081 | 1.117905 | 2.058457 | 1.603067 | 0.956505 | 0.894379 | 0.970643 |

| **HN49** | | | | | | | |
| --- | --- | --- | --- | --- | --- | --- | --- |
|  | **Blood** | **Primary T1** | **Primary T2** | **Secondary T1** | **Secondary T2** | **M1** | **M4** |
| **MYC** | -0.04971 | 0.296612 | 0.365791 | 0.340832 | 0.514306 | -0.20839 | 0.036795 |
| **COPYRATIO** | 0.966133 | 1.228256 | 1.288588 | 1.266487 | 1.428307 | 0.8655 | 1.025833 |

# Supplementary Figure

**Supplementary Figure S1**. Representative histological images illustrating (A) P53 staining, (B) reference slides with P53 staining and areas marked out for microdissection, (C) serial section used for LCM, (D) after LCM of tumor region (yellow), (E) after LCM of tumor (yellow) and basal layer (red) and (F) after LCM of squamous layer (blue), basal layer (red) and tumor cells (yellow). Scale bar: 500μm.


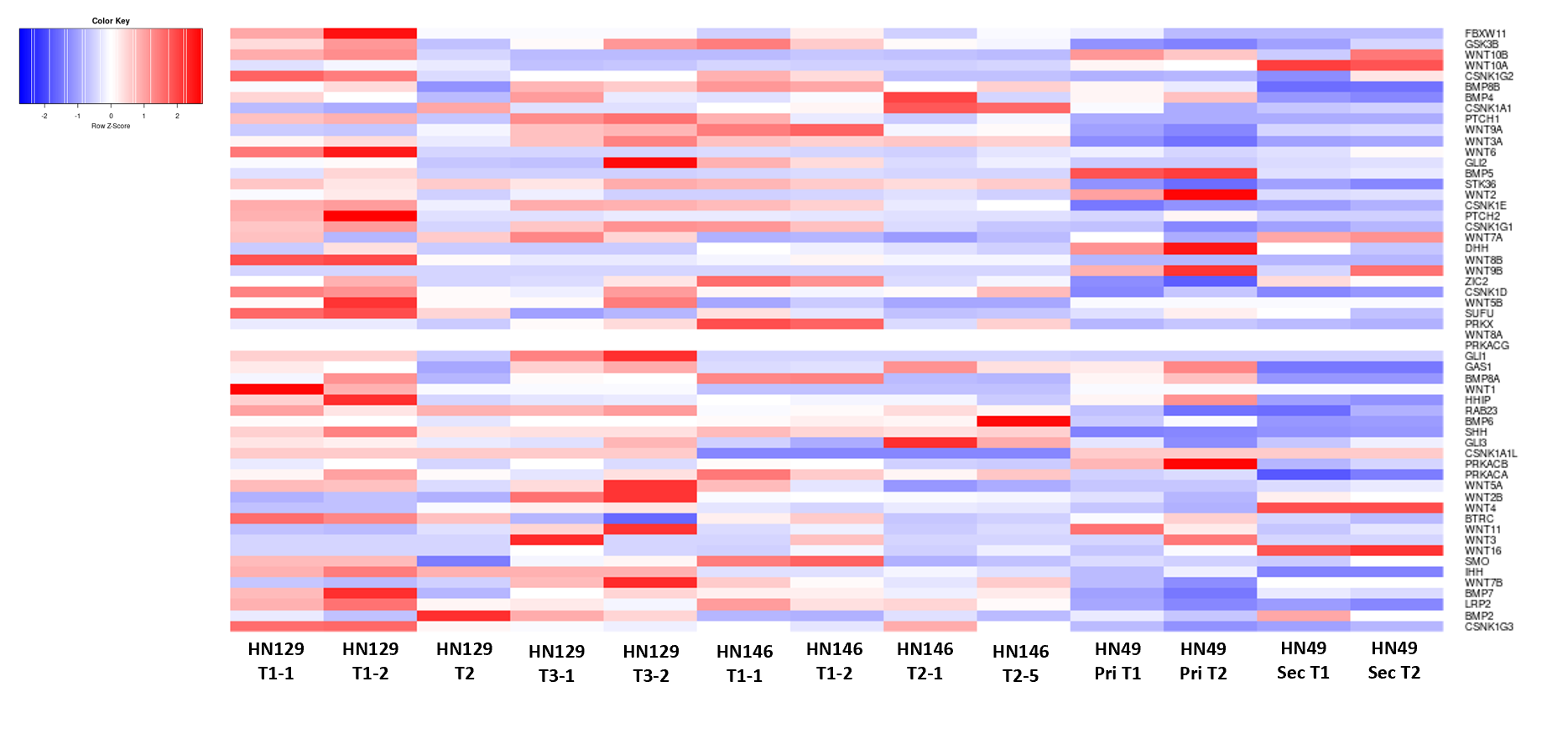


**Supplementary Figure S2.** Heatmap representing differentially expressed genes involved in Hedgehog signaling pathway across synchronous and metachronous tumors normalized to their respective normal mucosa.


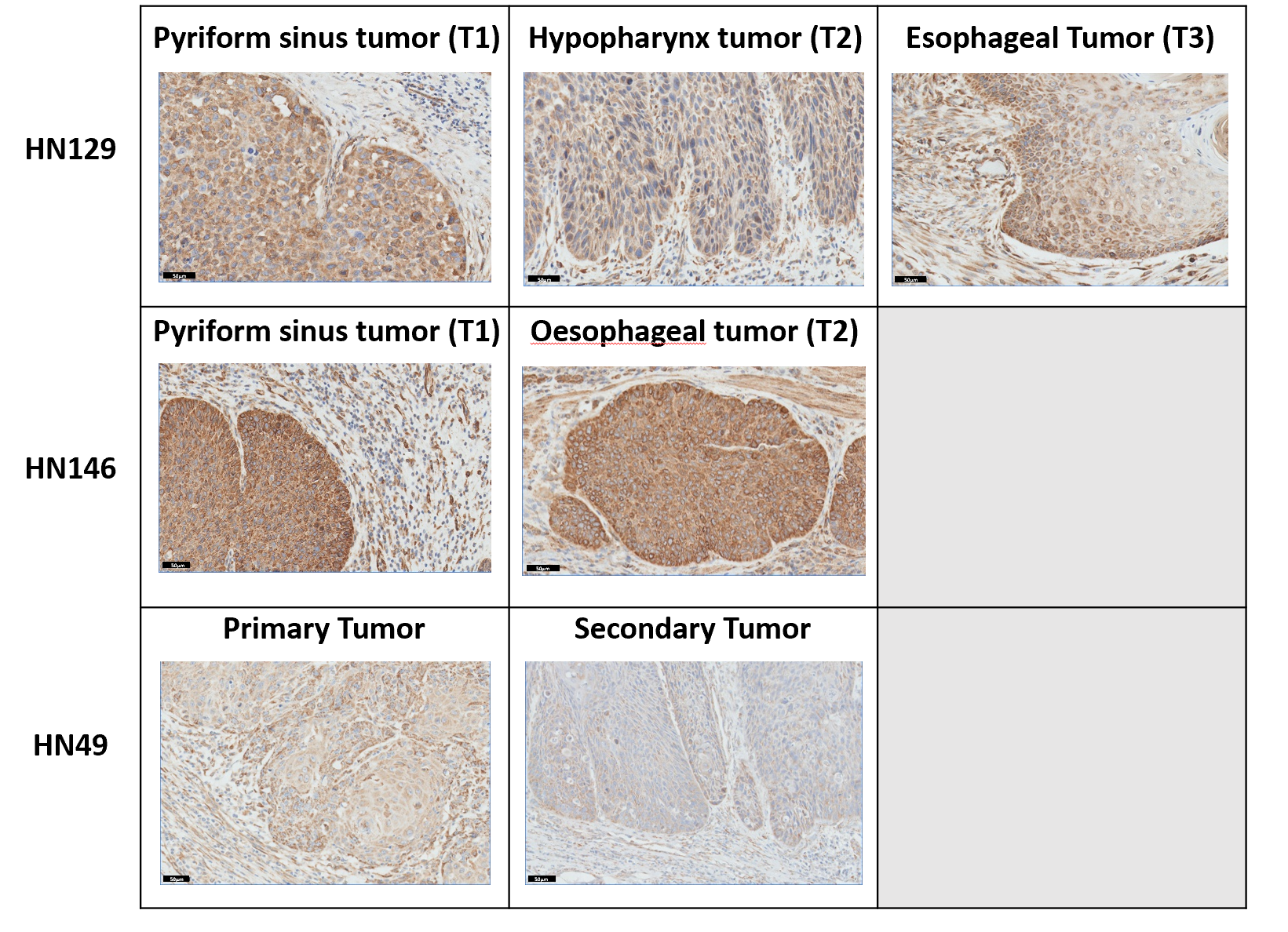


**Supplementary Figure S3**. Immunohistochemical (IHC) staining for Sonic Hedgehog of the sections from FFPE tumor samples derived from synchronous and metachronous tumors.

**Supplementary Figure S4**. (A) Representative IHC staining of Shh marker in a local cohort of HNSCC TMA core demonstrating (I) no expression and (II) overexpression. (B) Kaplan-Meier survival curve illustrating poorer prognosis in local cohort of patients with upregulated Hh signaling (*p*=0.014). (C) Kaplan-Meier survival curve illustrating poorer prognosis in TCGA HNSCC patients with upregulated Hh signaling (*p* = 0.015). *Shh, sonic hedgehog.*

**
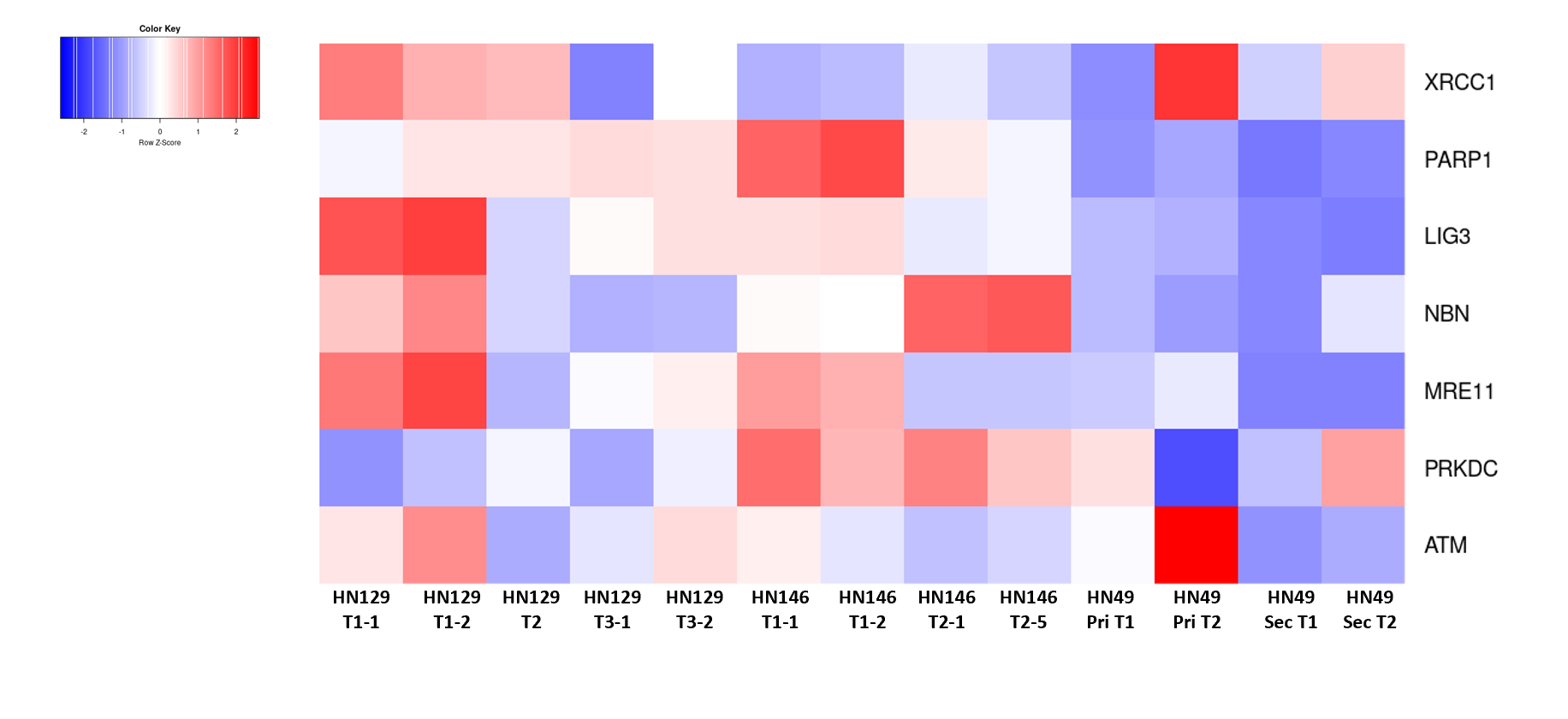
**

**Supplementary Figure S5.** Heatmap representing differentially expressed genes involved in PARP signaling across synchronous and metachronous tumors normalized to their respective normal mucosa.
